# The root hair defective phenotype of *Arabidopsis thaliana* Pol IV subunit mutant *nrpd1a-3* is associated with a deletion in RHD6

**DOI:** 10.17912/micropub.biology.000196

**Published:** 2019-12-12

**Authors:** Rakesh David, R. Daniel Kortschak, Iain Searle

**Affiliations:** 1 School of Biological Sciences, The University of Adelaide, The University of Adelaide and Shanghai Jiao Tong University Joint International Centre for Agriculture and Health, Adelaide, South Australia 5005, Australia; 2 Present address: ARC Centre of Excellence in Plant Energy Biology, The School of Agriculture, Food and Wine, University of Adelaide, Waite Campus, Adelaide, South Australia 5005, Australia

**Figure 1:**
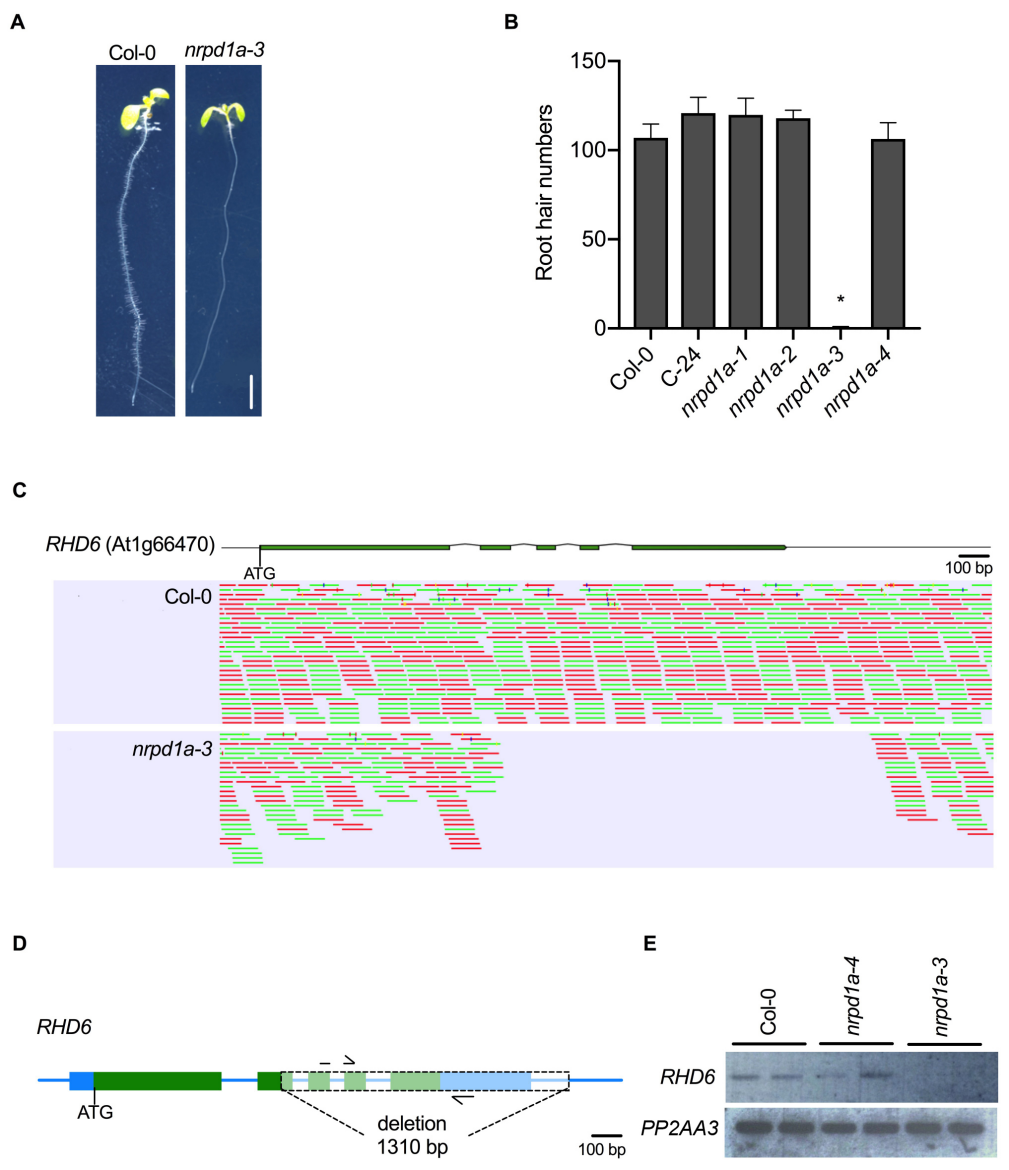
Root hair defective phenotype of *Pol IV* allele *nrpd1a-3* and identification of a DNA deletion in *Root Hair Defective 6* (*RHD6*). A. Root hair phenotype of Col-0 (wild type) and *NRPD1A* T-DNA mutant *nrpd1a-3*. Seedlings were grown in a vertical orientation for 7 days. Scale bar = 0.5 cm. B. Root hair numbers of Col-0, C-24, *nrpd1a-1*, *nrpd1a-2, nrpd1a-3* and *nrpd1a-4*. Alleles *nrpd1a-1* and *nrpd1a-2* are in the C-24 accession and alleles *nrpd1a-3* and *nrpd1a-4* are in the Col-0 accession. Between 18-20 seedlings roots were examined for each genotype, with an asterisk * denoting a significant difference between wildtype and mutant (p<0.005). Error bars show standard error. C. Top, shows a schematic representation of *RHD6* where the green boxes indicate exons and the intervening line black lines indicate introns. Below, is a genome browser view showing whole genome Illumina re-sequencing reads that aligned to *RHD6* in either Col-0 or *nrpd1a-3*. Green bars indicate sequence reads aligned to the top strand and red bars indicate reads aligned to the bottom strand. In *nrpd1a-3,* the region with no aligned sequence reads indicates the deleted region. Scale bar = 100 bp D. Schematic representation of *RHD6* showing the 1,310 bp deletion in *nrpd1a-3*. The arrows represent oligo-nucleotide primers that were used for semi-quantitative RT-PCR. E. *RHD6* transcripts was amplified from Col-0, *nrpd1a-3* and *nrpd1a-4* by semi-quantitative RT-PCR and the products were separated on an agarose gel. For each genotype two biological replicates were derived from a pool of 10 seedlings. *PP2AA3*was used as a positive control.

## Description

In eukaryotes, RNA and chromatin-based pathways control transposable elements (TE) to minimize the deleterious consequences of genetic invasion, transposition, mutation and chromosome instability (Matzke and Mosher, 2014). In higher plants, the multi-subunit nuclear RNA polymerase IV (Pol IV) specializes in transcribing the 24 nucleotide class of small RNAs that target TE’s for DNA cytosine methylation and silencing in the RNA-directed DNA methylation (RdDM) pathway (Herr *et al.*, 2005; Onodera *et al.*, 2005). In *Arabidopsis*, the Pol IV has two alternative subunits encoded by the NRPD1a and NRPD1b and a common subunit encoded by NRPD2A (Herr *et al.*, 2005; Kanno *et al.*, 2005; Onodera *et al.*, 2005; Pontier *et al.*, 2005). The null mutant for *NRPD1a* is defective in the RdDM pathway and also displays a late flowering phenotype under short day conditions (Pontier *et al.*, 2005; Eamens *et al.*, 2008).

Interestingly, we observed a root hair defective phenotype in the *nrpd1a-3* mutant allele which has not been previously reported (Fig. 1A). When grown on vertically oriented agar medium, *nrpd1a-3* seedlings lacked root hairs or defective root hair elongation when compared to the Col-0 wild type control which showed normal root hair distribution and length in the root maturation zone (Fig. 1A). Further investigation of other *NRPD1A* mutant alleles, *nrpd1a-1*, *nrpd1a-2,* in the C-24 accession, and *nrpd1a-4* in the Col-0 accession revealed that these alleles had a similar root hair number to the wild type controls (Fig. 1B). To investigate if the root hair defective phenotype in *nrpd1a-3* was a spurious event in our laboratory’s seed stock or an earlier event, we re-ordered the same mutant from the ABRC stock Centre and tested the root hair phenotype. We observed the same root hair defective phenotype in both *nrpd1a-3* seed stocks. We next crossed the *nrpd1a-3* mutant (Col-0) to wild type accession C-24, self-fertilized the F_1_to create a F_2_mapping population and mapped polymorphic DNA markers across the 5 chromosomes revealed the gene for the root hair defective phenotype was located between DNA markers ciw3 and F26B6 on chromosome 1 (Berendzen *et al.*, 2005). Next we whole-genome sequenced DNA from both Col-0 and *nrpd1a-3* using Illumina short read technology*,* and after GATK (McKenna *et al.*, 2010) and DELLY (Rausch *et al.*, 2012) analysis of the annotated gene models in the genetic window, we identified only one nucleotide mutation, a 1,310 bp deletion in *Root Hair Defective 6* (*RHD6*), in *nrpd1a-3* (Fig. 1C). *RHD6* is a bHLH transcription factor that positively regulates root hair initiation (Masucci and Schiefelbein, 1994) and loss of function mutations cause a root hair defective phenotype. Semi-quantitative RT-PCR showed *RHD6* mRNA was undetectable in the *nrpd1a-3* roots when compared to Col-0 wild-type and *nrpd1a-4* suggesting that the loss of *RHD6* was the likely candidate for the root hair defective phenotype observed in the *nrpd1a-3* mutant (Fig. 1D). We confirmed the 1,310 bp deletion that deleted part of exon 2, all of exons 3-5 and part of the 3’ UTR by PCR and Sanger sequencing. Together the genetic mapping and the undetectable *RHD6* mRNA transcript strongly suggests that the root hair defective phenotype only observed in the *nrpd1a-3* mutant background is caused by the deletion in *RHD6*. In the *Arabidopsis* research community, sometimes phenotypes caused by an unlinked mutation to a gene of interest have been incorrectly associated in the research field for many years (Enders *et al.*, 2015; Habets and Offringa, 2015), and so our discovery of a deletion in *RHD6* in *nrpd1a-3* will allow the community to not incorrectly associate the defective root hair phenotype with POLIV function.

## Reagents

For soil grown *A. thaliana* plants, seeds were germinated in soil and seedlings grown under halogen lights at 21°C under 16-hour light (130 µmoles/m^2^/sec) and 8-hour dark conditions as previously described (David *et al.*, 2017). For vertical plate experiments, seeds were chlorine gas sterilized as previously described (Burgess *et al.*, 2015) stratified at 4°C in the darkness and then transferred to grow under halogen lights of 16-hour light, 8-hour night conditions (130 µmoles/m^2^/sec). Seedlings were grown in a vertical orientation for 7 days on half-strength Murashige and Skoog (MS) medium containing 1% sucrose. Col-0 or C-24 accessions were used as wild-type controls. The *NRPD1A* two SALK T-DNA insertion lines in the Col-0 accessions for *NRPD1A* were *nrpd1a-3* (SALK_128428) and *nrpd1a-4* (SALK_083051). Mutants *nrpd1a-1* and *nrpd1a-2* are in the C-24 accession (Herr *et al.,* 2005). ImageJ was used to measure the number of root hairs longer than 2 mm from an image. Statistical analyses of the data were made using Student’s t-test.

**RT-PCR and PCR**

Total RNA was isolated from seedling roots using the Tri Reagent (Sigma Aldrich) as described by Wang *et al.,* 2017. Briefly, cDNA for semi-quantitative RT-PCR was synthesized using Superscript III kits as per the manufacturer’s recommendation (Invitrogen) from 2 mg of total RNA that was oligo(dT) primed. Genomic DNA for PCR or sequencing was isolated using the Dellaporta procedure (Dellaporta *et al.*, 1983) with small modifications as previously described from two-week-old seedlings grown on agar medium. RT-PCR and PCR reagents and thermal cycling conditions were previously described in David *et al.*, 2017.

**Table:** 

Gene name	Locus identifier	Application	Forward primer sequence	Reverse primer sequence
*RHD6*	AT1G14920	RT-PCR	CCAATGGCACCAAGGTTGATTT	TTTCCCCCGATATTATTACAACGTA
*PP2AA3*	AT1G13320	RT-PCR control	GGGCAATGCAGCATATAGTTC	TGGGTCTTCACTTAGCTCCAC
*RHD6*	AT1G66470	detection of deletion	AGGGCAACAACATGAGCTACGGC	TAAGAACACGTATCCCTAAT

**Whole-genome sequencing and Bioinformatic analysis**

Illumina sequencing libraries were prepared using NEBNext Ultra DNA library Prep kit as per the manufacturer’s recommendation and sequenced on the Illumina Hi-Seq system. Illumina sequence reads were trimmed by using TrimGalore! (Krueger, 2015), sequence quality assessed by using FastQC (Andrews, 2010), detection of nucleotide variation was performed by using DELLY(Rausch *et al.*, 2012)and GATK (McKenna *et al.*, 2010). All bioinformatic analysis were performed using default parameters. Sequence data from this article can be found in the SRA accession ID: PRJNA590836.

Gene sequence data from this article can be found in The Arabidopsis Information Resource (TAIR) under the following accession numbers: *NRPD1A* At1g63020; *RHD6* At1g66470 and *PP2AA3*At1g13320.

The *nrpd1a-3 rhd6-4* strain has been submitted to ABRC (Stock ID CS72356).

## References

[R1] Andrews S, 2010. FastQC: a quality control tool for high throughput sequence data. Available online at: https://www.bioinformatics.babraham.ac.uk/projects/fastqc/

[R2] Berendzen K, Searle I, Ravenscroft D, Koncz C, Batschauer A, Coupland G, Somssich IE, Ülker B (2005). A rapid and versatile combined DNA/RNA extraction protocol and its application to the analysis of a novel DNA marker set polymorphic between Arabidopsis thaliana ecotypes Col-0 and Landsberg erecta.. Plant Methods.

[R3] Burgess AL, David R, Searle IR (2015). Conservation of tRNA and rRNA 5-methylcytosine in the kingdom Plantae.. BMC Plant Biol.

[R4] David R, Burgess A, Parker B, Li J, Pulsford K, Sibbritt T, Preiss T, Searle IR (2017). Transcriptome-Wide Mapping of RNA 5-Methylcytosine in Arabidopsis mRNAs and Noncoding RNAs.. Plant Cell.

[R5] Eamens A, Vaistij FE, Jones L (2008). NRPD1a and NRPD1b are required to maintain post-transcriptional RNA silencing and RNA-directed DNA methylation in Arabidopsis.. Plant J.

[R6] Enders TA, Oh S, Yang Z, Montgomery BL, Strader LC (2015). Genome Sequencing of Arabidopsis abp1-5 Reveals Second-Site Mutations That May Affect Phenotypes.. Plant Cell.

[R7] Habets ME, Offringa R (2015). Auxin Binding Protein 1: A Red Herring After All?. Mol Plant.

[R8] Herr AJ, Jensen MB, Dalmay T, Baulcombe DC (2005). RNA polymerase IV directs silencing of endogenous DNA.. Science.

[R9] Kanno T, Huettel B, Mette MF, Aufsatz W, Jaligot E, Daxinger L, Kreil DP, Matzke M, Matzke AJ (2005). Atypical RNA polymerase subunits required for RNA-directed DNA methylation.. Nat Genet.

[R10] Krueger F. 2015. Trim Galore!: A wrapper tool around Cutadapt and FastQC to consistently apply quality and adapter trimming to FastQ files, with some extra functionality for MspI-digested RRBS-type (Reduced Representation Bisufite-Seq) libraries methylation. Available online at: https://www.bioinformatics.babraham.ac.uk/projects/trim_galore/

[R11] Masucci JD, Schiefelbein JW (1994). The rhd6 Mutation of Arabidopsis thaliana Alters Root-Hair Initiation through an Auxin- and Ethylene-Associated Process.. Plant Physiol.

[R12] Matzke MA, Mosher RA (2014). RNA-directed DNA methylation: an epigenetic pathway of increasing complexity.. Nat Rev Genet.

[R13] McKenna A, Hanna M, Banks E, Sivachenko A, Cibulskis K, Kernytsky A, Garimella K, Altshuler D, Gabriel S, Daly M, DePristo MA (2010). The Genome Analysis Toolkit: a MapReduce framework for analyzing next-generation DNA sequencing data.. Genome Res.

[R14] Onodera Y, Haag JR, Ream T, Costa Nunes P, Pontes O, Pikaard CS (2005). Plant nuclear RNA polymerase IV mediates siRNA and DNA methylation-dependent heterochromatin formation.. Cell.

[R15] Pontier D, Yahubyan G, Vega D, Bulski A, Saez-Vasquez J, Hakimi MA, Lerbs-Mache S, Colot V, Lagrange T (2005). Reinforcement of silencing at transposons and highly repeated sequences requires the concerted action of two distinct RNA polymerases IV in Arabidopsis.. Genes Dev.

[R16] Rausch T, Zichner T, Schlattl A, Stütz AM, Benes V, Korbel JO (2012). DELLY: structural variant discovery by integrated paired-end and split-read analysis.. Bioinformatics.

[R17] Wang D, Qu Z, Yang L, Zhang Q, Liu ZH, Do T, Adelson DL, Wang ZY, Searle I, Zhu JK (2017). Transposable elements (TEs) contribute to stress-related long intergenic noncoding RNAs in plants.. Plant J.

